# Bile Reflux is a Risk Factor for Metachronous Development in Patients with Early Gastric Cancer

**DOI:** 10.1055/a-2892-7590

**Published:** 2026-06-25

**Authors:** Hiroki Murai, Takuya Yamada, Yukihiro Kusumoto, Akane Namiki, Naoko Hayata, Akino Okamoto, Munehiro Ashida, Takafumi Tanimoto, Kohsaku Ohnishi, Tomohide Kurahashi, Motohiro Hirao, Atsushi Hosui, Naoki Hiramatsu

**Affiliations:** 1 Department of Gastroenterology and Hepatology 13824 Osaka Rosai HospitalSakaiJapan

**Keywords:** endoscopy upper GI tract, precancerous conditions & cancerous lesions (displasia and cancer) stomach, endoscopic resection (ESD, EMRc, …), diagnosis and imaging (inc chromoendoscopy, NBI, iSCAN, FICE, CLE)

## Abstract

**Background/Aims** The eradication of *Helicobacter pylori* reduces gastric cancer risk; however, the rate of metachronous development (MD) after endoscopic resection for early gastric cancer (EGC) is 1–4% annually. Hence, we evaluated various risk factors for MD after endoscopic submucosal dissection (ESD), specifically bile reflux, which has received limited clinical attention.

**Methods/Results** We retrospectively analyzed clinical data from 454 patients who underwent ESD for EGC between 2010 and 2018. MD was defined as the presence of a newly developed lesion more than 12 months after ESD. Bile reflux was assessed under standardized second-look endoscopy the day after ESD. During a median follow-up of 34.3 months, MD was observed in 45 patients (9.9%). Multivariate analysis revealed older age, severe gastric mucosal atrophy, xanthoma, and bile reflux as independent risk factors for MD. The 5-year MD rate was significantly greater in the bile reflux group than in the control group (24.2% vs. 11.2%, *p* = 0.01). Notably, in the bile reflux group, MD was significantly more frequently observed in the antrum or lower gastric body (61.1% vs. 29.6%, *p* = 0.036). Diabetes mellitus was identified as an independent risk factor for bile reflux.

**Conclusions** Bile reflux is a significant and independent risk factor for MD after ESD for EGC, particularly in gravity-dependent areas. Patients with bile reflux represent a high-risk subgroup requiring intensive endoscopic surveillance, such as every 6 months, to facilitate early detection of metachronous lesions.

## Introduction

Gastric cancer is the fourth leading cause of cancer-related death worldwide. 1 The eradication of *Helicobacter pylori* decreases the risk of gastric cancer; however, gastric cancer remains responsible for more than one million deaths annually worldwide. Improved endoscopic technologies have enabled the early detection of gastric cancer, and endoscopic submucosal dissection (ESD) provides results comparable to those of surgery while maintaining patient quality of life by preserving the stomach. However, the rate of metachronous gastric cancer (MGC) has been reported to be 1–4% per year even after ESD, 2 3 indicating a major clinical problem. Therefore, the identification of risk factors for MD is critical, and preventive methods are urgently needed. Previous studies have reported that older age, severe atrophic gastritis and multiple lesions are important risk factors for MD, 2 4 5 but most of these factors are either nonmodifiable or difficult to influence. There have been reports about the involvement of bile reflux in the development of residual gastric cancer or Barrett’s adenocarcinoma, but there are no reports about the risk factors for MD after ESD for early gastric cancer (EGC). Given that bile reflux was detected in the stomach via endoscopy in patients with MGC after ESD, we analyzed the risk factors for MD, particularly bile reflux. In this study, we revealed that bile reflux was related to MD after ESD for EGC and discussed host risk factors for bile reflux as well as methods for its prevention.

## Materials and Methods

### Patients

**Fig. 1:**
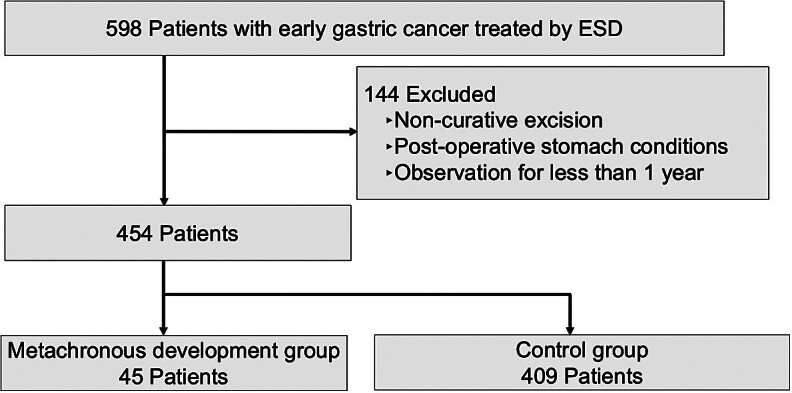
Flow chart illustrating the patient selection process and categorization into the control group and the metachronous development group. A total of 454 patients who underwent curative endoscopic submucosal dissection (ESD) for early gastric cancer were analyzed. MD, metachronous development; ESD, endoscopic submucosal dissection.

This retrospective observational study was conducted and reported in accordance with the Strengthening the Reporting of Observational Studies in Epidemiology guidelines. Among 598 patients who underwent first-time ESD for EGC at Osaka Rosai Hospital (Osaka, Japan) between January 2010 and December 2018, 454 patients were enrolled. Patients whose endoscopic resection was noncurative and those who were followed for less than 1 year after treatment were excluded ( Fig. 1 ). Curative endoscopic resection is pathologically defined as differentiated mucosal cancer without ulceration or with ulceration with a maximum diameter of less than 30 mm, no lymphatic or venous invasion, and negative horizontal and vertical margins. Follow-up included esophagogastroduodenoscopy at 6–12 month intervals after ESD, and MD was defined as the presence of a newly developed lesion in other parts of the stomach during endoscopic follow-up more than 12 months after curative endoscopic resection for EGC. Clinical characteristics, including age, sex, body mass index (BMI), drinking and smoking habits, and comorbidities, were collected. The status of *H. pylori* infection was determined on the basis of the presence of serum anti- *H. pylori* immunoglobulin G antibodies at the time of ESD. For patients who were positive for *H. pylori* and subsequently underwent eradication therapy after ESD, eradication success was determined using the 13C-urea breath test. Endoscopic findings, including the degree of gastric mucosal atrophy and the presence of intestinal metaplasia, xanthomas, bile reflux, or multiple lesions, were observed. Multiple simultaneous lesions were defined as the presence of two or more independent gastric lesions at the time of initial diagnosis or during first-time ESD. The study design was consistent with the principles of the Declaration of Helsinki. The study protocol was approved by the Institutional Review Board of Osaka Rosai Hospital.

### Evaluating Endoscopic Findings

**Fig. 2:**
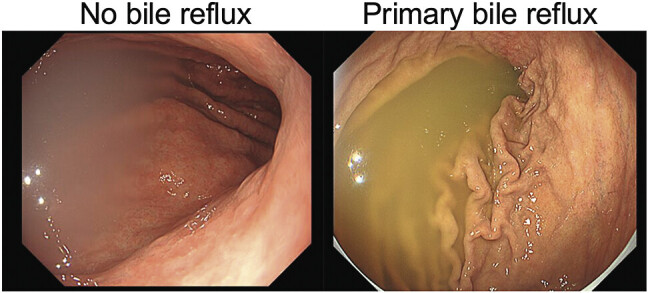
Representative endoscopic images of gastric findings. ( **A** ) Normal gastric mucosa without bile reflux. ( **B** ) Presence of bile reflux, characterized by a “yellowish-green mucous lake” in the stomach. Bile reflux was assessed via standardized second-look endoscopy the day after ESD.

The endoscopic video system used was EVIS LUCERA ELITE (Olympus, Tokyo, Japan), and the endoscopes used were GIF-HQ290 and GIF-H290Z (Olympus). Gastric mucosal atrophy was classified using the Kimura–Takemoto classification, with the closed type being mild atrophy and the open type being severe atrophy. Intestinal metaplasia was defined as the observation of grayish-white mucosa on white light imaging (WLI) or the presence of a light blue crest and a white opaque substance on image-enhanced endoscopy (IEE). Bile reflux was defined as the presence of a yellowish-green mucous lake in the stomach ( Fig. 2 ). This visual criterion is supported by a recent study by Wada et al., 6 which revealed that bilirubin concentrations greater than 1%—a clinically significant level—appear as distinct color changes on endoscopy. To ensure the reproducibility of our bile reflux assessment, we performed a standardized endoscopic procedure. Evaluation was conducted the day after ESD. The assessment was based on the observation of gastric fluid immediately upon insertion of the endoscope into the stomach; this protocol specifically excludes contamination from endoscope-induced gagging. All patients fasted for a specified duration and were confirmed to have started proton pump inhibitor (PPI) or P-CAB therapy prior to ESD to ensure a consistent pharmacological environment. Bile reflux assessments were conducted by multiple expert endoscopists in a blinded manner.

### Statistical Analysis

Statistical analyses were performed with Prism ver. 8.4.2 for Mac (GraphPad, San Diego, CA, USA) and SPSS software version 24 (IBM Corporation, Armonk, NY, USA). Mann–Whitney U tests were performed to assess differences between unpaired groups. Correlations were assessed using the Pearson product–moment correlation coefficient. The Kaplan–Meier method and log-rank test were used to analyze differences in MD rates. Univariate and multivariate Cox proportional hazards model analyses were performed to identify factors associated with MD. Hazard ratios and 95% confidence intervals (CIs) are shown. Univariate and multivariate logistic regression analyses were performed to identify factors associated with bile reflux risk. Odds ratios and 95% CIs are shown. A *p* value < 0.05 was considered to indicate statistical significance unless otherwise indicated.

## Results

### Older Age, Severe Gastric Mucosal Atrophy, Xanthoma, and Bile Reflux were Risk Factors for MD after ESD for EGC

The baseline characteristics of the patients enrolled in this study are shown in Table 1 . The median age was 74 years; 341 patients were male, and 113 were female. Seventy-six patients were smokers, and 145 were drinkers. Hypertension was observed as a comorbidity in 265 patients (58%), dyslipidemia in 157 patients (35%), diabetes mellitus in 79 patients (17%), chronic kidney disease in 71 patients (16%), and stroke in 42 patients (9%). *H. pylori* infection was found in 217 patients (48%). The median observation period was 34.3 months. The endoscopic findings of the patients included in this study are shown in Table 2 . Severe gastric mucosal atrophy was present in 216 patients (48%), intestinal metaplasia in 156 patients (34%), xanthoma in 138 patients (30%), bile reflux in 115 patients (25%), and multiple simultaneous lesions in 48 patients (11%). MD was observed in 45 patients (9.9%) during the observation period. The results of the univariate and multivariate Cox proportional hazards model analyses of factors associated with MD are shown in Table 3 . In the univariate analysis, older age ( *p* < 0.01), smoking status ( *p* = 0.04), severe atrophy ( *p* < 0.01), intestinal metaplasia ( *p* < 0.01), xanthoma ( *p* < 0.01), and bile reflux ( *p* = 0.01) were identified as significant risk factors for MD. The multivariate analysis revealed that older age (HR 2.05, 95% CI 1.11–3.80; *p* = 0.02), severe atrophy (HR 2.74, 95% CI 1.16–6.48; *p* = 0.02), xanthoma (HR 2.11, 95% CI 1.17–3.82; *p* = 0.01), and bile reflux (HR 1.98, 95% CI 1.07–3.66; *p* = 0. 03) were significant factors.

**Table 1:** Clinical characteristics of 454 patients who underwent endoscopic submucosal dissection (ESD) for early gastric cancer (EGC). This table presents the baseline clinical data, including age, sex, comorbidities, and *H. pylori* status, for the total cohort and the subgroup that developed metachronous lesions.

Factor	All ( *n* = 454)	Control ( *n* = 409)	MD ( *n* = 45)
Age (years)	74 (36–91)	74 (36–91)	78 (60–86)
Sex: female/male (% male)	113/341 (75%)	101/308 (75%)	12/33 (73%)
BMI (% ≥25)	22.8 (21%)	22.8 (20%)	22.9 (29%)
Smoking: no/yes (% yes)	378/76 (17%)	345/64 (16%)	33/12 (27%)
Alcohol: no/yes (% yes)	309/145 (32%)	280/129 (32%)	29/16 (36%)
HT: no/yes (% yes)	189/265 (58%)	177/232 (57%)	12/33 (73%)
DL: no/yes (% yes)	297/157 (35%)	271/138 (34%)	26/19 (42%)
DM: no/yes (% yes)	375/79 (17%)	341/68 (17%)	34/11 (24%)
CKD: no/yes (% yes)	383/71 (16%)	344/65 (16%)	39/6 (13%)
Stroke: no/yes (% yes)	412/42 (9%)	370/39 (10%)	42/3 (6%)
*H. pylori* infection status: negative/positive (% positive)	237/217 (48%)	214/195 (48%)	23/22 (49%)
Follow-up period (month)	34 (1–122)	35 (2–122)	30 (1–101)

MD: metachronous development, HT: hypertension, DL: dyslipidemia, DM: diabetes mellitus, CKD: chronic kidney disease, *H. pylori* : *Helicobacter pylori* .

**Table 2:** Endoscopic findings of 454 patients who underwent ESD for EGC. This table details the prevalence of gastric mucosal atrophy, intestinal metaplasia, xanthoma, bile reflux, and simultaneous multiple lesions identified during the endoscopic assessment.

Factor	All ( *n* = 454)	Control ( *n* = 409)	MD ( *n* = 45)
Gastric mucosal atrophy: mild/severe (% severe)	238/216 (48%)	227/182 (45%)	11/34 (76%)
Intestinal metaplasia: absent/present (% present)	298/156 (34%)	281/128 (31%)	17/28 (62%)
Xanthoma: absent/present (% present)	315/138 (30%)	293/116 (28%)	23/22 (49%)
Bile reflux: absent/present (% present)	339/115 (25%)	312/97 (24%)	27/18 (40%)
Multiple lesions: absent/present (% present)	406/48 (11%)	367/42 (10%)	39/6 (13%)

MD: metachronous development.

**Table 3:** Univariate and multivariate Cox proportional hazards analyses of risk factors associated with metachronous development. This table shows the hazard ratios and statistical significance of various factors, with older age, severe atrophy, xanthoma, and bile reflux identified as independent predictors of metachronous development.

		Univariate analysis			Multivariate analysis		
Factor	Category	Hazard ratio	95% CI	*p* -Value	Hazard ratio	95% CI	*p* -Value
Age (y)	<75/≥75	2.46	1.35–4.50	<0.01	2.05	1.11–3.80	0.02
Sex	Female/male	0.86	0.45–1.67	0.67			
BMI (kg/m ^2^ )	<25/≥25	1.6	0.85–3.01	0.15			
Smoking	No/yes	2	1.03–3.88	0.04	1.7	0.87–3.34	0.12
Alcohol	No/yes	1.18	0.64–2.18	0.59			
HT	No/yes	1.81	0.94–3.51	0.08			
DL	No/yes	1.25	0.69–2.26	0.46			
DM	No/yes	1.23	0.62–2.44	0.55			
CKD	No/yes	0.8	0.34–1.90	0.61			
Stroke	No/yes	0.99	0.31–3.22	0.99			
*H. pylori* infection status	Negative/positive	0.89	0.50–1.60	0.7			
Gastric mucosal atrophy	Mild/severe	3.76	1.90–7.43	<0.01	2.74	1.16–6.48	0.02
Intestinal metaplasia	Absent/present	3.31	1.81–6.06	<0.01	1.81	0.85–3.88	0.13
Xanthoma	Absent/present	2.19	1.22–3.94	<0.01	2.11	1.17–3.82	0.01
Bile reflux	Absent/present	2.12	1.16–3.86	0.01	1.98	1.07–3.66	0.03
Multiple lesions	Absent/present	1.88	0.79–4.49	0.15			

HT: hypertension, DL: dyslipidemia, DM: diabetes mellitus, CKD: chronic kidney disease, *H. pylori* : *Helicobacter pylori.*

### The Presence of Bile Reflux Doubled the 5-Year Rate of MD after ESD for EGC

**Fig. 3:**
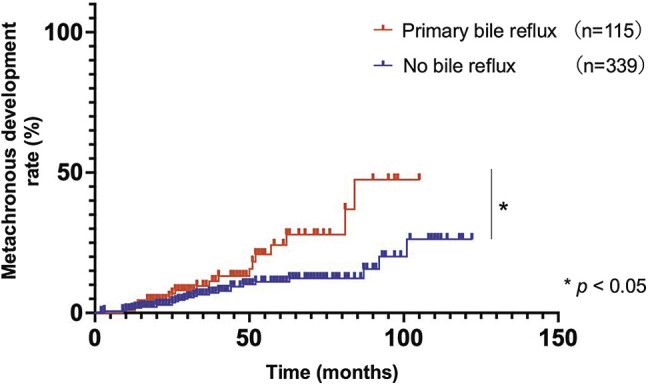
Kaplan–Meier analysis of metachronous development (MD) rates after endoscopic submucosal dissection (ESD) for early gastric cancer (EGC). The cumulative incidence of MD was compared between the bile reflux group (red line, *n* = 115) and the control group without bile reflux (blue line, *n* = 339). The incidence of MD was significantly greater in the bile reflux group than in the control group, with 5-year MD rates of 24.2% and 11.2%, respectively. Statistical significance was determined using the log-rank test ( *p* = 0.01). MD, metachronous development; ESD, endoscopic submucosal dissection; EGC, early gastric cancer.

The MD rates after ESD for EGC in the bile reflux group and the control group are presented as Kaplan–Meier curves in Fig. 3 . The overall 3- and 5-year MD rates were 7.4% and 14.5%, respectively. The rates were 9.6% and 24.2% in the bile reflux group and 6.6% and 11.2% in the control group, respectively, with significantly higher MD rates in the bile reflux group ( *p* = 0.01).

### Metachronous Development was Significantly more Frequent in the Antrum or Lower Gastric Body in the Bile Reflux Group

We analyzed the anatomical distribution of the 45 metachronous lesions identified during the follow-up period. Metachronous development (MD) in the antrum or lower gastric body—areas most susceptible to concentrated bile exposure due to gravity—occurred in 61.1% (11/18) of patients in the bile reflux group compared with 29.6% (8/27) of patients in the control group. This difference was statistically significant ( *p* = 0.036), underscoring the localized impact of bile reflux on gastric carcinogenesis ( Table 4 ).

**Table 4:** Anatomical distribution of metachronous lesions in patients with and without bile reflux. This table compares the proportion of lesions occurring in the antrum or lower gastric body versus other areas, highlighting a significantly higher incidence in gravity-dependent areas in the bile reflux group.

Anatomical distribution	All ( *n* = 45)	Others ( *n* = 27)	Bile reflux ( *n* = 18)
Middle or upper gastric body and fundus	26 (57.8%)	19 (70.4%)	7 (38.9%)
Antrum or lower gastric body	19 (42.2%)	8 (29.6%)	11 (61.1%)

### Diabetes Mellitus was an Independent Risk Factor for Bile Reflux

We then examined host risk factors for bile reflux. Bile reflux was observed in 115 of 454 patients (25.3%), and we compared the background factors of patients in the bile reflux group with those in the control group **(** Table 5 ). Univariate and multivariate logistic regression analyses revealed the risk factors associated with bile reflux ( Table 6 ). In the univariate analysis, smoking ( *p* < 0.05) and diabetes mellitus ( *p* < 0.01) were identified as significant risk factors. Only diabetes mellitus was identified as an independent risk factor for bile reflux by multivariate analysis (OR 2.11, 95% CI 1.26–3.54; *p* < 0.01).

**Table 5:** Comparison of clinical characteristics between patients in the bile reflux group and the control group. This table shows the differences in background factors, such as smoking habits and comorbidities, between patients with and without bile reflux.

Factor	Others ( *n* = 339)	Bile reflux ( *n* = 115)
Age (years)	74	74
Sex: female/male (% male)	85/254 (74.9%)	28/87 (75.7%)
BMI (% ≥25)	22.9 (24.8%)	22.5 (17.4%)
Smoking: no/yes (% yes)	289/50 (14.7%)	89/26 (22.6%)
Alcohol: no/yes (% yes)	230/109 (32.2%)	79/36 (31.3%)
HT: no/yes (% yes)	149/190 (56.0%)	40/75 (65.2%)
DL: no/yes (% yes)	224/115 (33.9%)	73/42 (36.5%)
DM: no/yes (% yes)	290/49 (14.5%)	85/30 (26.1%)
CKD: no/yes (% yes)	285/54 (15.9%)	98/17 (14.8%)
Stroke: no/yes (% yes)	307/32 (9.4%)	105/10 (8.7%)
*H. pylori* infection status: negative/positive (% positive)	181/158 (46.6%)	56/59 (51.3%)

HT: hypertension, DL: dyslipidemia, DM: diabetes mellitus, CKD: chronic kidney disease, *H. pylori* : *Helicobacter pylori* .

**Table 6:** Univariate and multivariate logistic regression analyses of factors associated with bile reflux. This table presents the odds ratios for factors predisposing patients to bile reflux, with diabetes mellitus identified as a significant independent risk factor.

		Univariate analysis			Multivariate analysis		
Factor	Category	Odds ratio	95% CI	*p* -Value	Odds ratio	95% CI	*p* -Value
Age (y)	<75/≥75	1.03	0.67–1.57	0.91			
Sex	Female/male	1.04	0.64–1.70	0.88			
BMI (kg/m ^2^ )	<25/≥25	0.64	0.37–1.10	0.11			
Smoking	No/yes	1.69	1.00–2.87	<0.05	1.71	1.00–2.92	0.05
Alcohol	No/yes	0.96	0.61–1.52	0.87			
HT	No/yes	1.47	0.95–2.28	0.09			
DL	No/yes	1.12	0.72–1.74	0.61			
DM	No/yes	2.09	1.25–3.50	<0.01	2.11	1.26–3.54	<0.01
CKD	No/yes	0.92	0.51–1.65	0.77			
Stroke	No/yes	0.91	0.43–1.92	0.81			
*H. pylori* infection status	Negative/positive	1.21	0.79–1.84	0.38			

HT: hypertension, DL: dyslipidemia, DM: diabetes mellitus, CKD: chronic kidney disease, *H. pylori* : *Helicobacter pylori* .

## Discussion

Although advances in endoscopic technology and instruments have made safe and curative resection of EGC possible, MGC remains an important issue. Therefore, the identification of risk factors for MGC is critical, and preventive methods are urgently needed. G Mori et al. reported that successful *H. pylori* eradication is important for reducing gastric cancer risk and that male sex, severe gastric mucosal atrophy, and multiple gastric cancers are independent risk factors for MGC. 2 Choe et al. reported that severe atrophic gastritis or the presence of intestinal metaplasia is related to a significantly increased incidence of MGC. 4 Although the methods used to analyze the risk factors for MGC vary among studies, older age and severe gastric mucosal atrophy are considered common risk factors for MGC, which is consistent with the results of our study. In our study, *H. pylori* infection at the time of ESD was not a risk factor for MGC. Chang Seok Bang et al. reported that *H. pylori* eradication after ESD for EGC reduces the incidence of MGC, 7 whereas Chen HN et al. reported that *H. pylori* eradication was less effective in patients after ESD for EGC than in the general population. 8 Although whether *H. pylori* eradication after ESD for EGC is effective at preventing MGC remains unknown, we administered *H. pylori* eradication treatment to almost all patients with *H. pylori* infection after ESD in our hospital. In our study, bile reflux was identified as an independent risk factor for MD. Although some reports indicate that bile reflux is a risk factor for residual gastric cancer or Barrett’s adenocarcinoma, to our knowledge, this is the first study to reveal bile reflux as a risk factor for MGC. A possible mechanism of carcinogenesis by bile reflux is chronic gastric mucosal damage associated with exposure to bile acids and the induction of inflammatory mediators such as IL-8 and COX-2, which are involved in the activation of a cascade of various cytokines associated with subsequent cancer growth and progression. 9 10 We then examined the risk factors and preventive methods for bile reflux. Numerous factors have been reported to be associated with bile reflux gastritis, including age, sex, body type, lifestyle habits, diabetes, gallbladder diseases, psychological factors, and *H. pylori* infection, but diabetes is considered one of the most important risk factors. 10 11 A considerable proportion of diabetic patients suffer from gastroparesis. Long-term hyperglycemia can induce disorders of the autonomic nervous system, which reduce the tension of the stomach and slow gastric peristalsis, thus leading to delayed gastric emptying and abnormal gastro-pyloric-duodenal dynamics that prolong the period during which bile remains in the stomach and contribute to the occurrence of bile reflux. 12 Diabetic microvascular lesions also reduce blood flow to the gastric mucosa, causing gastric peristalsis to slow. 13 From these perspectives, treatments that reduce bile reflux and therefore decrease metachronous gastric cancer are needed. The first goal is to improve glycemic control among diabetic patients and prevent neurovascular disease due to persistent hyperglycemia. The second goal is to initiate medication treatment to reduce gastroparesis and gastric mucosal damage. Although there is no official uniform therapeutic regimen for bile reflux, ursodeoxycholic acid (UDCA), hydrotalcite, PPIs, and prokinetic agents have been widely accepted for its management. 14 Indeed, several reports have shown that the administration of mosapride citrate hydrate and UDCA improved gastroparesis and reduced gastric mucosal damage. 14 15 16 17 Mosapride citrate hydrate promotes gastrointestinal motility and gastric emptying, while UDCA plays a dual role in protecting the gastric mucosa and accelerating the excretion of endogenous bile acids. Given that these drugs have relatively few side effects, their administration to patients with bile reflux may be a valuable strategy to mitigate the risk of MD. Furthermore, our findings directly informed our post-ESD clinical practice. For EGC patients with bile reflux, we increased the frequency of endoscopic surveillance from annually to every 6 months to facilitate the early detection of high-risk metachronous lesions.

The limitations of this study include the following. First, it was a single-center, retrospective study. Second, the influence of potential confounders, such as medications, gastric motility factors (e.g., pyloric function and duodenal motility), and post-ESD anatomical changes, on the endoscopic visibility of bile was not measured in this study. We were unable to measure the concentration of bile acids in the gastric mucus directly. However, this may also be advantageous: the risk of MGC can be evaluated using WLI alone, without the need for IEE, magnification, or other specialized techniques.

In conclusion, older age, severe gastric mucosal atrophy, xanthoma, and bile reflux are independent risk factors for MD after ESD for EGC. In particular, bile reflux is a significant indicator of a high-risk gastric environment for carcinogenesis. These findings suggest that patients with bile reflux represent a high-risk subgroup that requires stringent endoscopic surveillance, potentially with shortened intervals, such as every 6 months, to facilitate the early detection of metachronous lesions.

**Abbreviations**
BMI: body mass index
BRG: bile reflux gastritis
CKD: chronic kidney disease
DL: dyslipidemia
DM: diabetes mellitus
EGC: early gastric cancer
ESD: endoscopic submucosal dissection
HT: hypertension
*H. pylori*: *Helicobacter pylori*
IEE: image-enhanced endoscopy
IRB: Institutional Review Board
LBC: light blue crest
MGC: metachronous gastric cancer
MD: metachronous development
P-CAB: potassium-competitive acid blocker
PPI: proton pump inhibitor
UDCA: ursodeoxycholic acid
WLI: white light imaging
WOS: white opaque substance
